# Lists of monosyllables for logoaudiometric tests: elaboration, content validation and search for equivalence

**DOI:** 10.1590/2317-1782/20212021057

**Published:** 2022-01-07

**Authors:** Ana Valéria de Almeida Vaucher, Lidiéli Dalla Costa, Anaelena Bragança de Moraes, Isabela Hoffmeister Menegotto, Maristela Julio Costa

**Affiliations:** 1 Universidade Federal de Santa Maria – UFSM - Santa Maria (RS), Brasil.; 2 Universidade Federal de Ciências da Saúde de Porto Alegre – UFCSPA - Porto Alegre (RS), Brasil.

**Keywords:** Hearing, Speech Perception, Audiometry Speech, Validation Study, Psychometrics

## Abstract

**Purpose:**

Develop new lists of monosyllables for conducting logoaudiometric tests in Portuguese, perform content validation, considering ear side and education and check the equivalence between the lists.

**Methods:**

Were selected 125 monosyllables with different syllabic structures, which were submitted to the content validation process, which included judgment on familiarity, organization of lists, recording of material and auditory recognition. After content validation, the monosyllable lists were subjected to equivalence research, in order to obtain evidence of reliability for the proposed test instrument.

**Results:**

Five lists with 25 monosyllables were elaborated and analyzed for content, of these, four lists were validated. There was no statistically significant difference between the responses obtained in the right and left ears. The education of the subjects did not influence the recognition of words. As for the equivalence search, it was found that two lists were equivalent, one not equivalent, but similar and one list was different from the others, and then excluded.

**Conclusion:**

Two monosyllable lists were validated for content and considered equivalent, with the same level of difficulty between them, and one list was considered similar, which can be used as training to apply the test on the audiological battery. The validated lists were not influenced by ear and education.

## INTRODUCTION

Speech audiometry is a fundamental procedure for basic audiological evaluation. Its results assist in the confirmation of tonal thresholds, the measurement of auditory skills for detection and recognition of speech, the confirmation of diagnostic hypotheses, topodiagnosis, detection of functional or non-organic hearing loss and malingering, and also in the indication and evaluation of the benefits of sound amplification^(1)^.

One of the tests applied in the performance of speech audiometry is the Speech Recognition Percentage Index (SRPI), which evaluates the ability of the listener to recognize speech stimuli, at an intensity that allows the best possible performance^(2)^. Such intensity can vary between 20 and 60 dB NS, but generally, the presentation of the stimuli occurs at 40 dB NS^(3,4)^ or at the most comfortable level^(5)^.

Several stimuli can be used in the evaluation of the patient's ability to recognize speech, from meaningless syllables, monosyllabic words and even sentences. The stimuli used the most in the evaluation of speech recognition include lists of monosyllables^(6)^, as they enable investigation the individual's ability to recognize speech stimuli only based on their auditory ability, since they provide the least possible clues, ensuring the sensitivity of the test^(7)^.

In order to ensure scientific rigor, ensuring that the evaluation instrument used is valid and reliable, the ideal is for each language to have its own materials with speech stimuli for the performance of speech audiometry, and that these materials have recognized psychometric characteristics of validity and reliability^(2)^.

In Brazil, some lists of monosyllabic words have already been elaborated for speech recognition evaluation purposes^(4,5,8-11)^, which have been made available for use in clinical practice, some in live speech and others in recorded material. However, there is no information in the literature consulted about the realization of psychometric studies in the elaboration of these lists.

Thus, to fill this gap, in a PhD research project, new lists of monosyllables for the Portuguese language were elaborated, and content and construct validation measures and equivalence study on the lists were obtained, the results of which were presented in different articles, and the construct validation article has already been published^(12)^.

Thus, the purpose of this research was to describe the development of new lists of monosyllables for carrying out speech audiometry tests in the Portuguese language and also the to validate the content, considering the side of the ear and education level, and the verification of equivalence between the lists.

## METHODS

The research was approved by the Research Ethics Committee (REC) of the institution under number 13932513.1.0000.5346, meeting all ethical standards of conduct in research with human beings, in accordance with the Guidelines and Regulatory Standards for Research involving Human Beings (Resolution 466/12 of the National Health Council). All subjects read and signed the Informed Consent Form (ICF), consenting to their participation.

To carry out the study, both in the content validation stage and in the list equivalence study, an Interacoustics brand audiometer, model AC 33, with supra-aural earphone, model TDH-39, was used. The speech stimuli were presented in recorded form, using a Toshiba brand CD player, coupled to the audiometer.

### Content validation

The content validation was carried out in five stages, as described below:

In the ***first stage*** , the monosyllables were selected, to begin the content validation process. The words were extracted from local and state newspapers and also from a book from the speech therapy area, which contained words to be used in speech therapy. 125 monosyllabic, stressed or unstressed words belonging to any grammatical class were included, namely: noun, adjective, verb, pronoun, adverb, numeral, preposition, conjunction and interjection. As for the syllable structure, 60 words (48%) had the consonant-vowel-glide structure (**CVG**^1^, e.g. mau = ['maw], tem = ['tej]), 37 words (29.6%) were consonant-vowel-consonant (**CVC**^2^, e.g. por = ['por]), 15 words (12%) were consonant-vowel (**CV**, e.g. pá = ['pa]), 5 words (4%) were consonant-consonant-vowel-consonant (**CCVC,** e.g. três = ['tres]), 5 words (4%) were vowel-glide (**VG,** e.g. eu = ['ew]) and 3 words (2.4%) were consonant-vowel-glide-consonant (**CVGC,** e.g. meus = ['mews]). Monosyllables with other syllabic structures, words indicative of people's names and pseudowords were excluded.

In the ***second stage*** , there was the judgment of the familiarity of the words, which were sent by e-mail, to nine expert judges for analysis, four of which are active in the area of Phonetics/Phonology and five in the area of Audiology, as well as eight non-specialist judges working in other areas of knowledge. The judges were selected by convenience.

The judges evaluated each word on the list in relation to its familiarity, following a five-point Likert scale, classifying them as: Extremely Familiar (EF), Very Familiar (VF), Familiar (F), Slightly Familiar (SF) and Not Familiar (NF).

The data were analyzed based on the Content Validity Ratio (CVR) (Equation 1),


CVR= ne−N2N2 (1)

considering, as follows: $n_{e}$ = number of evaluators who judged the items as essential and N = 17: total number of evaluators who judged the items.

For the calculation of the CVR, the classification of items suggested by Lawshe (1975)^(13)^ was considered, which classified the items as “essential”, “useful, but not essential” and also as “not necessary”. The words evaluated as PE, VF and F, were considered the essential words; those judged as SF, were considered to be useful, but not essential, and the words analyzed as NF were considered not necessary, and able to be excluded from the set of words previously selected.

According to the author^(13)^, in order to avoid the values agreed between the judges being obtained at random and considering the sample obtained in this research (at least 13 judges considered a certain item as essential), the minimum value of the CVR should be 0.54, demonstrating that it would be unlikely that the agreement by the judges was random.

In the ***third stage*** , the words considered familiar, that is, the essential ones according to the calculation of the CVR, formed a word bank that was used in the elaboration of the preliminary version of the new lists of monosyllables, consisting of five lists with 25 monosyllables in each list, named as lists L1, L2, L3, L4 and L5, which were digitally recorded according to ISO 8253-3:2012.

In the ***fourth stage*** , 40 individuals were selected by convenience, aged between 18 and 44 years, right-handed, with different education levels, normal hearing (hearing thresholds ≤ 25 dB HL in frequencies from 250 Hz to 8,000 Hz, according to pure tone audiometry), without alterations in the external auditory canal and middle ear based on visual inspection and tympanometry curve, respectively, or without other impairments, such as alteration of comprehension and/or oral emission. Those subjects who met the selection criteria and signed the informed consent form participated in the research.

These individuals were considered as normal-hearing judges, to whom the lists of words were presented and, as a response, to they were asked to repeat the words as they understood them. The level of presentation was 40 dB NS.

In the ***fifth* and last stage**, the auditory judgment of the words was carried out through descriptive and qualitative analysis of the errors produced by the individuals. Further, the errors presented by ear were compared, using the Mann-Whitney U test (two independent samples, non-parametric test), and also considering the education level, using the Kruskal-Wallis test (four independent samples, non-parametric test). A 95% confidence interval was adopted.

After this stage, the reorganization of the words in the lists was carried out. During this reorganization, the words incorrectly emitted by more than one subject were excluded, which were considered non-random errors, and furthermore, the fifth list was excluded due to the reduction in the number of available words and because it presented the most heterogeneous composition. Thus, four lists remained, with 25 monosyllables validated for content, ready to be evaluated for equivalence between each other.

### Equivalence study of the lists

After content validation of the lists of monosyllables, they were submitted to an equivalence study.

In this stage, 60 individuals were selected by convenience, aged between 18 and 24 years, considering the other inclusion criteria mentioned in the fourth stage.

In this study, we chose to use a fixed noise, known as the *speech noise* type, at the intensity of 30 dB HL, in order to level the hearing of all participants and also neutralize any possible interference of body or environmental noise that could interfere with the performance of the individuals evaluated, as well as considering a level of presentation that did not cause discomfort and/or tiredness. This strategy of using a fixed noise to verify the equivalence of the lists of monosyllables had already been applied by other researchers^(14)^.

From that moment on, a pilot study was carried out, seeking to determine the signal/noise (SNR) ratio that would enable the obtainment of recognition scores that varied between 40 and 60%. This strategy was used in order to reduce the variability of the performance of the individuals evaluated, in addition to avoiding the “floor effect” or “celling effect”^(15)^, which occurs when performance is 0% or 100%.

Thus, considering the issues mentioned above, for the pilot study, the lists of words were presented in the presence of a fixed noise, presented ipsilaterally (speech and noise in the same ear) in different SNR ratios. The -1 dB HL ratio was the condition in which the scores came closest to the expected range

Based on the pilot study, the equivalence study of the lists was based on the performance of the other individuals, presenting the words at the level of 29 dB HL and noise at 30 dB HL (SNR ratio of -1 dB). The order of presentation of the lists was randomized, and 30 of them heard the words in the right ear and the other 30 in the left ear.

The subjects’ responses for each word were considered as either incorrect, assigning the value of “0”, or correct, with the value of “1”. Using these values, the percentage of correct answers of words per list was obtained, indicating the variability within each list. The subject’s total score per list was also considered, multiplying the number of correct answers by each subject by four to obtain the word recognition percentage index, that is, the subject’s performance per list.

To analyze the performance presented by the subjects according to the side of the ear, the Kruskal-Wallis test (variables with non-normal distribution, non-parametric test) was used.

Next, the percentages of correct answers of the words per list were analyzed to obtain the intelligibility percentage of each list, and then the analysis of the variance of the words per list was performed, applying the ANOVA test (variables with normal distribution, parametric test).

Finally, the subjects’ scores were analyzed per list. As variables with non-normal distribution, non-parametric tests were used: the Friedman test and analysis of variance between the lists, and the Wilcoxon test to identify the differences between the lists. Descriptive statistical analysis of some variables was also performed to complement the data analysis.

A significance level of 5% (p-value ≤ 0.05) was adopted. The statistically significant results were marked with an asterisk (*). Statistical analyses were performed using the *Statistica 9.1* software.

## RESULTS

In relation to content validation, the analysis of judgments of familiarity for the 125 words selected, based on the calculation of the Content Validity Ratio (CVR), revealed that all words presented CVR ≥ 0.529, and were considered essential (extremely familiar, very familiar and familiar), with no need to exclude any word at this stage (Table 1).

**Table 1:** Distribution of essential monosyllables (n=125) in the lists drawn up based on the Content Validity Ratio Calculation

**N Words (%)**	**N evaluators** **(N, %)**	**CVR**
**59 (47.2%)**	17 (100%)	1
**25 (20%)**	16 (94.11%)	0.882
**12 (9.6%)**	15 (88.23%)	0.764
**04 (3.2%)**	14 (82.35%)	0.647
**25 (20%)**	13 (76.47%)	0.529

Caption: N = number; CVR = Content Validity Ratio

Regarding the errors produced by subjects when performing the auditory recognition of the words, it is observed that there was no statistically significant difference when comparing the errors presented by the subjects who heard the words in the right ear and those who heard the words in the left ear, which allowed the data to be grouped for analysis of only the errors produced by the subjects (Chart 1).

**Chart 1:** Auditory recognition of lists of monosyllables by subjects of different education levels

Education level	N	Words produced with errors					N of errors per ear		p-value	N of words with errors by education level	p-value	Number of subjects with recognition errors
		L1	L2	L3	L4	L5	RE	LE				
Higher Education	10	0	bem	0	0	0	0	1	0.735	1	0.113	1
High School	10	giz	tom	deu	0	ai	2	2		4		4
Complete Elementary School	10	for	viu	0	0	0	2	0		2		2
Incomplete Elementary School	10	giz	sei	0	pai	ai zaz	3	2		5		4
Total	40	3	4	1	1	3	7	5		12		11

Analysis of errors per ear: Mann-Whitney U test (p-value = 0.735)

Analysis of errors by education level: Kruskal-Wallis test (p-value = 0.113)

Statistically significant value (p-value ≤0.05)

Caption: L1 = List 1; L2 = List 2; L3 = List 3; L4 = List 4; L5 = List 5; RE = right ear; LE = left ear; N = number

**Figure 1:**
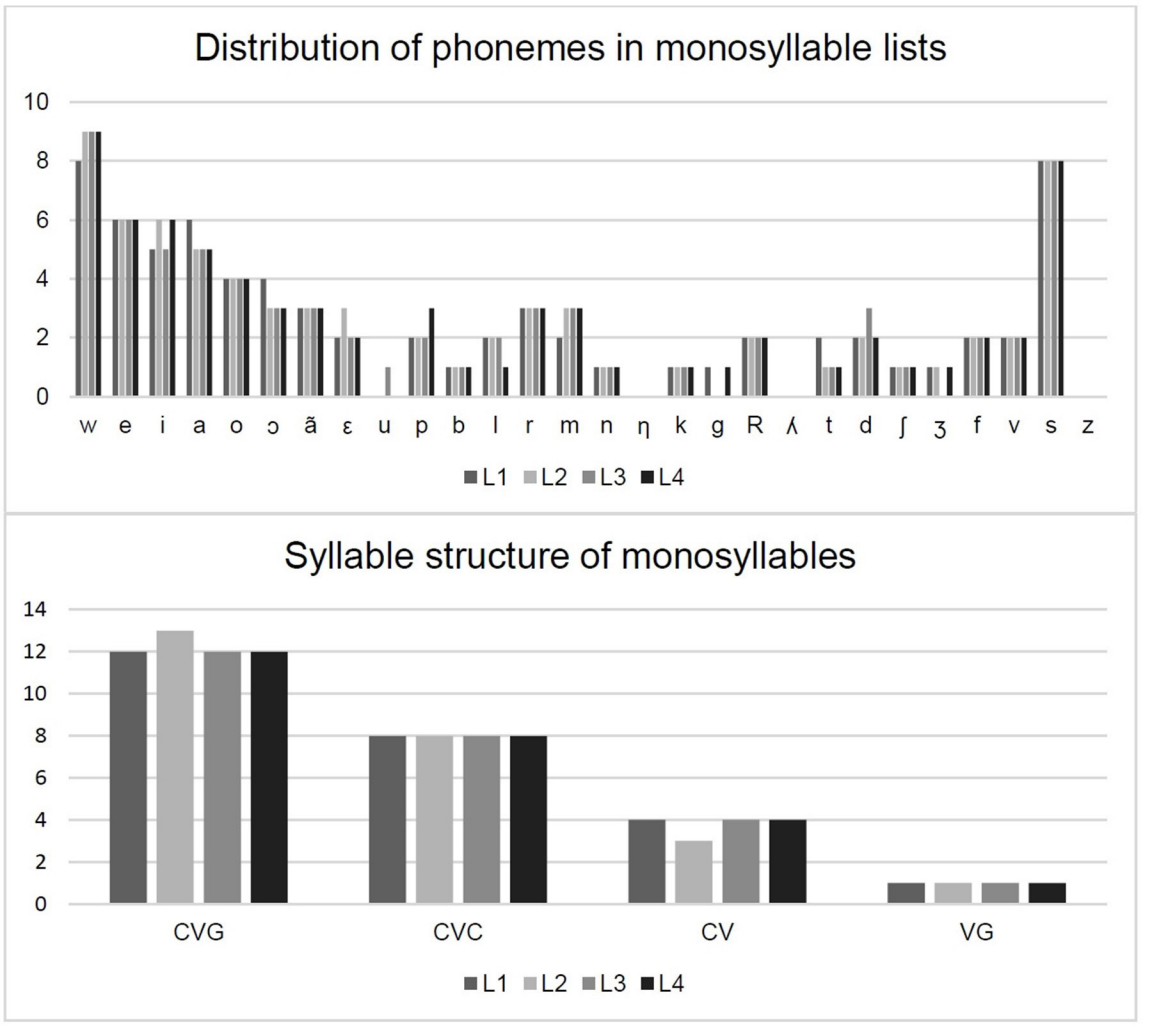
Presentation of the final version of the lists of monosyllables elaborated and validated regarding the content - distribution of phonemes per list and relation of syllable patterns

The content validation resulted in four lists of monosyllables (Figure 1). It can be noted that most of the phonemes that make up the lists are represented by the vowels and glides and also by the fricative phoneme /s/ that was present in several positions in the syllables. There are no monosyllables in any of the lists with the phonemes /ɲ/, /ʎ/ and /z/. However, the classes of these phonemes are represented by the phonemes /m/ and /n/, /l/ and /s/, respectively.

Caption: L1 = List 1; L2 = List 2; L3 = List 3; L4 = List 4

With regard to the equivalence study of the lists, using the Kruskal-Wallis test it was possible to observe that the p-values (p = 1,000 for all analyses performed) obtained by comparing the performance presented by the subjects in the different lists, per ear, were not statistically significant, which showed no difference in performance according to the side of the ear. Thus, the following analyses of the subjects’ responses were performed together.

As for the descriptive measures related to the percentages of correct answers (Table 2), on one side, the intelligibility percentages of the words per list are represented, analyzing the intralist variability, and on the other, the performance of the subjects in the recognition of the words per list of monosyllables is represented, analyzing the interlist variability.

**Table 2:** Descriptive analysis of the data, expressed as a percentage of correct answers, presenting the results according to intralist and interlist variability

**Analysis of words per List**				**List**	**Subjects' performance per List**			
L1	L2	L3	L4		L1	L2	L3	L4
25	25	25	25	**N**	60	60	60	60
48.33	48.00	55.73	51.93	**Mean (%)**	48.33	48.00	55.73	51.93
45	50	60	50	**Median (%)**	48	48	56	52
1.66	8.33	10	8.33	**Minimum (%)**	36	32	44	40
98.33	91.66	96.66	95.00	**Maximum (%)**	64	68	72	72
28.33	25.00	36.66	31.66	**Lower Quartile (%)**	44	44	52	44
70.00	61.66	75.00	75.00	**Upper Quartile (%)**	52	52	60	56
29.545	26.369	25.088	27.565	**Standard deviation (%)**	7.036	6.628	6.744	7.741

Thus, considering the percentage of correct answers of the words in the lists, it can be seen that list L3 presented the highest average of correct answers, appearing to be an easier list. Furthermore, it can be observed that the average percentage of correct answers of the words per list is very similar in lists L1 and L2, indicating that these would be the most homogeneous lists. On the other hand, lists L3 and L4 present a different average from lists L1 and L2. However, no statistically significant difference was found in this sense as per the ANOVA test (p-value = 0.722).

**Figure 2:**
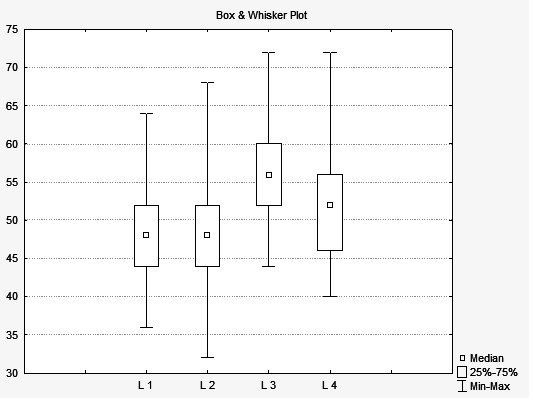
Representation of the comparison between the performance of the subjects in the recognition of the monosyllables of lists L1, L2, L3 and L4. Representation of the median and the minimum and maximum values in relation to the percentage of correct answers of the subjects per list of monosyllables - Interlist variability Friedman test – multiple dependent samples, paired by subject; p-value = 0.00000* *Statistically significant value (p-value ≤ 0.05) Caption: Median = Median; Min = Minimum; Max = Maximum

In turn, when analyzing the results of the comparison of the scores of the subjects between the lists of monosyllables, which show the median percentage of the subjects’ correct answers per list, it is possible to visually identify the similarity between the lists L1 and L2, while at the same time observing a slight similarity between L1 and L2 with L4, a lot of difference between L1 and L2 in relation to L3, and also a slight similarity between L3 and L4 (Figure 2). The Friedman test showed a statistical difference between the lists, based on the performance of the subjects.

Furthermore, the result of the statistical analysis with the application of the Wilcoxon test shows that list L1 does not differ from L2, and that there was a statistically significant difference when comparing these with lists L3 and L4 and comparing L3 and L4 with each other (Table 3).

**Table 3:** Comparison between the lists of monosyllables based on the subjects’ performance in the recognition of the words per list

**Lists**	**p-value**
L1 = L2	0.776
L1 ≠ L3; L4	≤ 0.01^*^
L2 ≠ L3; L4	≤ 0.001*
L3 ≠ L4	≤ 0.003*

Wilcoxon test – dependent samples

* Statistically significant value (p-value ≤ 0.01)

## DISCUSSION

Content validation is an essential step in the process of construction and adaptation of measurement instruments^(13)^. It is related to the planning of the test, at which time a set of relevant and representative items of the content to be evaluated is organized. Thus, in relation to the speech tests, it is initially necessary to consider the selection of words, given that they have particularities specific to each language, and should be standardized and tested in the subjects^(2)^.

The syllabic structures of the 125 words selected for the instrument proposed in this study, as described in the methodology, are in accordance with the characteristics of the Portuguese language, maintaining the syllabic structures of the language. A survey of the frequency of occurrence of the syllable patterns of Brazilian Portuguese, regardless of the number of syllables in the words, obtained based on written words from a dictionary, indicates that the CV structure is the most frequent in the language, followed by the syllables of the CVG or CVC, V, VC and CCV type, with the CCVC and CCVCC syllables being less frequent^(16,17)^. It should also be considered that in the Portuguese Language, there is a restricted number of monosyllables^(17)^, therefore, there are few consonant and vowel combinations that produce meaningful words.

In this study, the inclusion of words in the lists prioritized their familiarity, to the detriment of phonetic balance (Table 1), as according to the literature^(18)^, the familiarity of a word is dependent on the frequency of use in the language and is also related to improvement in the intelligibility of words. In addition, the choice of familiar words would minimize the effect of educational differences between the subjects tested, thereby justifying and reinforcing the criterion chosen for the selection of words.

Furthermore, when choosing the words to form the lists for this study, a representative sample of the sounds of the language was kept, with a distribution of the phonemes among the lists, which was as uniform as possible. This practice was corroborated in the literature consulted, demonstrating that the phonetic balance is of secondary importance, since the most used and significant words are those that allow better speech recognition^(7,19)^, once again reinforcing the need for the inclusion of familiar words.

Of the speech tests elaborated in Brazil with monosyllabic words, Sá^(8)^ has elaborated phonetically balanced lists. Lacerda^(10)^ chose words whose phonetic material was representative of the language, yet without performing rigorous phonetic balancing. It was also ensured that the words were familiar, that they were part of the usual vocabulary, that they represented concrete nouns, that they were accessible to people from different cultural levels and that the lists had the same degree of difficulty between each other.

Pen and Mangabeira-Albernaz^(9)^ and Russo and Santos^(5)^ used familiar words, but did not clarify how they selected the words. Chaves et al.^(11)^ and Roll et al.^(4)^ relied on the criteria proposed by Lacerda^(10)^ for choice of words. Chaves et al.^(11)^ drew up lists of 25 words and 25 pseudowords (monosyllabic and disyllabic) and Roll et al.^(4)^ drew up two lists of monosyllables, one with 25 items and the other with 50.

When analyzing the influence of education level on the errors produced by the evaluated subjects (Chart 1), no statistically significant difference was found, but it was identified that 11 subjects presented errors in the auditory recognition of 12 words.

Most of the errors presented by the subjects were considered to be random, as they were isolated errors. However, some can be explained by the acoustic-articulatory characteristics of the phonemes and also by the familiarity, according to the meaning that the word assumes for the listener, as mentioned by some authors^(4,20)^.

It was also observed that exchanges were more frequent in consonant sounds than with vowels, which is similar to the results obtained in another study^(21)^, justifying that speech intelligibility is dependent on consonant sounds, which represent a contribution of 60%, with vowels contributing only 40%.

The equivalence studies of lists of monosyllables present very varied strategies. Thus, in the analysis of the equivalence of the Mandarin monosyllable lists^(22)^, for example, the authors fixed the level of the speech signal at 10 dB NS from the tritone average of the frequencies of 500, 1000 and 2000 Hz, to obtain recognition scores between 40 and 60%. Further, in another study with lists of Mandarin monosyllables^(14)^, each subject was tested with all lists being applied at different sequences and with different presentation levels (-5, 0, 5, 10 and 15 dB HL). Another study, which investigated psychometric functions in the presence of noise^(23)^, used four SNR ratios (-7, -2, 3 and 8 dB), keeping the noise fixed at 72 dB SPL, seeking the 50% point. These researchers, each with their own strategies, were able to evaluate the equivalence of their lists, and confirm that the performance of the subjects was significantly influenced by the SNR ratio chosen.

Thus, because we did not find any consensus strategy in the literature, and because this is a new material, we used the presentation of word lists in the presence of different noise levels in a pilot study to check the equivalence between the lists. This allowed us to determine the appropriate SNR ratio to investigate the speech recognition index avoiding the occurrence of the “floor” or “ceiling” effect^(15)^. These effects should be avoided, as they cause the loss of information in the observed data due to the possibility that the true scores are above the minimum or maximum limits observed in the threshold, where it is no longer possible to measure the variable. These are considered censored data, that is, data that is only partially known^(24)^.

It is believed that the variety of strategies used by different researchers is not a problem, as it is known that many factors are involved in this type of analysis, such as the equipment used, calibration, application method, composition and familiarity of the words, voice of the speaker, type of noise to be used, and the characteristics of the individuals evaluated, among others. It should be noted that at this stage of the research, it was sought to establish a relative measure, consisting of comparing the different lists between each other, maintaining the conditions of invariable tests and very similar individual characteristics of the subjects evaluated, with strict inclusion and exclusion criteria, and having only the subjects as the main variable, thereby avoiding compromising the results obtained as much as possible.

The intralist variability was expressed in this study through the intelligibility percentage of each word per list (Table 2), thus providing information on the variation of intelligibility of the items on the lists.

It can be seen that in the four lists, none of the words had 0% or 100% correct answers, and statistically there is a balance between the intelligibility of the words in each list. This result is in accordance with the assumptions relating to the criteria to be observed in the preparation of speech materials^(25)^.

In this study, it is clear that lists L1 and L2 are very similar (Table 2 and Figure 2), confirmed by the mean and median values, which represent a measure of central tendency for the speech recognition scores presented by the subjects. The values found in the quartiles also suggest that lists L1 and L2 are equivalent. List L4 presented the third value for mean and median, being similar to lists L1 and L2 but not equivalent to them. List L3 was considered the easiest list among the four proposed lists.

It is important to note that obtaining the equivalence of different word lists is a complex task, and difficult to obtain even with all the criteria based on the literature for the selection of words, preparation of lists and recording of such^(2,19,25)^, which can be attributed to a number of factors. These factors are related to the intelligibility of the words on the lists that encompass the phonetic construction, the familiarity of the word, the phonetic environment of the word and, above all, the recording characteristics of the speech tests, including the recording techniques and the characteristics of the speaker^(25,26)^.

Considering the results obtained (Figure 2 and Table 3), it was observed that, although not presenting statistical equivalence with lists L1 and L2, list L4 enabled similar scores and presented homogeneity in relation to the variation of the intelligibility of the items. Thus, it is suggested that it be used as a training list, which is extremely useful in order to familiarize the individual with the test, when necessary.

List L3 will be excluded, although it was considered homogeneous in relation to the variation in the intelligibility of the items, as it presented very different scores in relation to the other lists.

After content validation and the equivalence study, the lists from this research were renamed in alphabetical order and therefore named as the Training List, L1 and L2 (Appendix 1).

It should be noted that the psychometric measures obtained in this study are valid for the lists recorded as they are. If they are re-recorded by another person or even by the same person, if they are presented by live voice, or if there is a new organization of words originating other lists, new psychometric measures will need to be obtained. This premise is corroborated in the consulted literature^(23,27,28)^.

It is believed that the elaboration and validation of the proposed material in digital format, according to the recommendations of the literature, will allow the realization of the SRPI in a more precise manner, reducing variability and increasing the reproducibility of the results obtained in the live voice evaluation.

## CONCLUSION

Two lists of monosyllables were validated as to content and considered equivalent, and one list was considered similar and could be used as training, forming a set of three lists developed in the Brazilian Portuguese language and digitally recorded, which may be part of the speech audiometry test battery to be applied in clinical routine and in research, after its validation and publication. The validated lists were not influenced according to the side of the ear and education level.

## Footnotes

1 In this syllable pattern, the nasal and lateral liquid consonants in the final coda position and the second vowel of the oral and nasal diphthongs were considered as glides, due to their phonetic characteristics.

2 The consonants at the end of the syllable, in the final coda position, are represented by the consonants /r/ and /s/.

## Appendix 1 Lists of monosyllables for speech audiometry tests

**Table:**

	LIST TRAINING	L1	L2
1	não	bar	dez
2	só	dó	fim
3	véu	eu	pá
4	paz	for	ver
5	lei	gás	mas
6	dom	mil	dói
7	fã	céu	seu
8	eu	vão	lã
9	bis	ter	gel
10	rio	jaz	rês
11	mar	nem	chão
12	pé	pós	nó
13	gol	dei	sei
14	foz	tão	por
15	cão	som	mau
16	ser	rói	fiz
17	já	leu	tom
18	meu	voz	viu
19	xis	cai	eu
20	tem	mim	cós
21	dor	pau	mão
22	sal	sã	réu
23	rim	ré	lar
24	pai	lá	sai
25	vez	fez	bem
