# Supplementary material for: Lists of monosyllables for logoaudiometric tests: elaboration, content validation and search for equivalence
Source: Codas. 2022 Jan 7;34(3):e20210057. doi: 10.1590/2317-1782/20212021057 (PMC9851190; doi:10.1590/2317-1782/20212021057)
Supplement: Translation

# Translation: Listas de monossílabos para testes logoaudiométricos: elaboração, validação de conteúdo e pesquisa de equivalência

**Authors:** Ana Valéria de Almeida Vaucher, Lidiéli Dalla Costa, Anaelena Bragança de Moraes, Isabela Hoffmeister Menegotto, Maristela Julio Costa

**Affiliations:** 1 Universidade Federal de Santa Maria – UFSM - Santa Maria (RS), Brasil.; 2 Universidade Federal de Ciências da Saúde de Porto Alegre – UFCSPA - Porto Alegre (RS), Brasil.

**Keywords:** Audição, Percepção da Fala, Audiometria da Fala, Estudo de Validação, Psicometria

## Abstract

**Objetivo:**

Elaborar novas listas de monossílabos para realização de testes logoaudiométricos na Língua Portuguesa, realizar a validação de conteúdo, considerando lado da orelha e escolaridade e verificar a equivalência entre as listas.

**Método:**

Foram selecionados 125 monossílabos com diferentes estruturas silábicas, os quais foram submetidos ao processo de validação de conteúdo, que incluiu o julgamento quanto à familiaridade, organização das listas, gravação do material e reconhecimento auditivo. Após a validação de conteúdo, as listas de monossílabos foram submetidas à pesquisa de equivalência, a fim de obter evidências de fidedignidade para o instrumento de teste proposto.

**Resultados:**

Cinco listas com 25 monossílabos foram elaboradas e analisadas quanto ao conteúdo, destas, quatro listas foram validadas. Não houve diferença estatisticamente significante entre as respostas obtidas nas orelhas direitas e esquerdas. A escolaridade dos sujeitos não influenciou o reconhecimento das palavras. Quanto à pesquisa de equivalência, verificou-se que duas listas foram equivalentes, uma não equivalente, porém semelhante e uma lista foi diferente das demais, e então excluída.

**Conclusão:**

Duas listas de monossílabos foram validadas quanto ao conteúdo e consideradas equivalentes, com igual nível de dificuldade entre si e uma lista foi considerada semelhante, podendo ser utilizada como treino para aplicação do teste na bateria audiológica. As listas validadas não sofreram influência segundo o lado da orelha e escolaridade.

## INTRODUÇÃO

A logoaudiometria é um procedimento fundamental da avaliação audiológica básica. Seus resultados auxiliam na confirmação dos limiares tonais, na mensuração das habilidades auditivas de detecção e reconhecimento de fala, na confirmação de hipóteses diagnósticas, topodiagnóstico, detecção de perdas auditivas funcionais ou não orgânicas e nos quadros de simulação, e ainda, na indicação e avaliação dos benefícios da amplificação sonora^(1)^.

Um dos testes aplicados na realização da logoaudiometria é o Índice Percentual de Reconhecimento de Fala (IPRF), o qual avalia a habilidade do ouvinte para reconhecer os estímulos de fala, em uma intensidade que permita o melhor desempenho possível^(2)^. Tal intensidade pode variar entre 20 e 60 dB NS, mas geralmente, a apresentação dos estímulos ocorre à 40 dB NS^(3,4)^ ou no nível de máximo conforto^(5)^.

Vários estímulos podem ser utilizados na avaliação da habilidade do paciente em reconhecer a fala, desde sílabas sem sentido, palavras monossilábicas e até sentenças. Dentre os estímulos mais utilizados na avaliação do reconhecimento de fala, estão as listas de monossílabos^(6)^, pois permitem investigar a capacidade do indivíduo para reconhecer os estímulos de fala apenas com base na sua habilidade auditiva, uma vez que fornecem o mínimo de pistas possíveis, garantindo a sensibilidade do teste^(7)^.

A fim de garantir rigor científico, assegurando que o instrumento de avaliação utilizado é válido e confiável, o ideal é que cada língua possua seus próprios materiais com estímulos de fala para realização da logoaudiometria e que esses materiais apresentem características psicométricas de validade e confiabilidade reconhecidas^(2)^.

No Brasil, já foram elaboradas algumas listas de palavras monossilábicas para fins de avaliação do reconhecimento de fala^(4,5,8-11)^, que foram disponibilizadas para serem utilizadas na prática clínica, algumas à viva voz, outras em material gravado. No entanto, não há informações, na literatura consultada, sobre a realização de estudos psicométricos na elaboração dessas listas.

Sendo assim, para preencher essa lacuna, em uma pesquisa de Doutorado, foram elaboradas novas listas de monossílabos para a Língua Portuguesa, e obtidas medidas de validação de conteúdo, construto e pesquisa de equivalência das listas, cujos resultados foram apresentados em diferentes artigos, sendo que o artigo de validação de construto já foi publicado^(12)^.

Dessa forma, a proposta desta pesquisa foi descrever a elaboração de novas listas de monossílabos para realização de testes logoaudiométricos na Língua Portuguesa e também a validação de conteúdo, considerando lado da orelha e escolaridade e a verificação da equivalência entre as listas.

## MÉTODO

A pesquisa foi aprovada pelo Comitê de Ética em Pesquisa (CEP) da instituição sob número 13932513.1.0000.5346, atendendo todas as normas éticas de conduta em pesquisa com seres humanos, de acordo com as Diretrizes e Normas Regulamentadoras de Pesquisa envolvendo Seres Humanos (Resolução 466/12 do Conselho Nacional de Saúde). Todos os sujeitos leram e assinaram o Termo de Consentimento Livre e Esclarecido (TCLE), consentindo a sua participação.

Para execução do estudo, tanto na etapa de validação de conteúdo, quanto na pesquisa de equivalência das listas, foi utilizado um audiômetro marca Interacoustics, modelo AC 33, com fone supra-aural modelo TDH-39. Os estímulos de fala foram apresentados na forma gravada, utilizando-se um aparelho de *CD player,* marca Toshiba, acoplado ao audiômetro.

### Validação de conteúdo

A validação de conteúdo foi realizada em cinco etapas, descritas a seguir:

Na ***primeira etapa*** ocorreu a seleção dos monossílabos, dando início ao processo de validação de conteúdo. As palavras foram extraídas de jornais de circulação local e estadual e também de um livro da área fonoaudiológica, que continha palavras para serem utilizadas em fonoterapia. Foram incluídas 125 palavras monossilábicas, tônicas ou átonas, pertencentes a qualquer classe gramatical, a saber: substantivo, adjetivo, verbo, pronome, advérbio, numeral, preposição, conjunção e interjeição. Quanto à estrutura de sílaba, 60 palavras (48%) tinham a estrutura consoante-vogal-glide (**CVG**^1^, ex. mau = ['maw], tem = ['tej]), 37 palavras (29,6%) consoante-vogal-consoante (**CVC**^2^, ex. por = ['por]), 15 palavras (12%) consoante-vogal (**CV**, ex. pá = ['pa]), 5 palavras (4%) consoante-consoante-vogal-consoante (**CCVC,** ex. três = ['tres]), 5 palavras (4%) vogal-glide (**VG,** ex. eu = ['ew]) e 3 palavras (2,4%) consoante-vogal-glide-consoante (**CVGC,** ex. meus = ['mews]). Foram excluídos os monossílabos com outras estruturas silábicas, os vocábulos indicativos de nomes de pessoas e as pseudopalavras.

Na ***segunda etapa*** , ocorreu o julgamento da familiaridade dos vocábulos, que foram enviados via correio eletrônico, para nove juízes especialistas para análise, sendo que quatro destes tinham atuação na área da Fonética/Fonologia e cinco na área da Audiologia, e também para oito juízes não especialistas com atuação em outras áreas de conhecimento. A seleção dos juízes se deu por conveniência.

Os juízes avaliaram cada vocábulo da lista em relação à sua familiaridade, seguindo uma escala Likert de cinco pontos, classificando-os como: Extremamente Familiar (EF), Muito Familiar (MF), Familiar (F), Pouco Familiar (PF) e Nada Familiar (NF).

Os dados foram analisados com base na Razão de Validade de Conteúdo (RVC) considerando (Equação 1),


RVC= ne−N2N2 (1)

sendo, $n_{e}$ = número de avaliadores que julgaram os itens como essenciais e N = 17: número total de avaliadores que realizaram o julgamento dos itens.

Para a realização do cálculo da RVC foi considerada a classificação de itens sugerida por Lawshe^(13)^, o qual classificou os itens em essenciais, em úteis, mas não essenciais e também somente em não essenciais, os vocábulos avaliados como EF, MF e F, foram considerados os vocábulos essenciais; os julgados como PF, foram considerados como sendo vocábulos úteis, mas não essenciais, e os vocábulos analisados como NF, foram considerados não essenciais e passíveis de serem excluídos do conjunto de palavras previamente selecionadas.

Segundo o autor^(13)^, para evitar que os valores de concordância entre os juízes fossem obtidos ao acaso, o valor mínimo do RVC, considerando a amostra obtida nesta pesquisa (no mínimo 13 juízes consideraram determinado item como essencial), deveria ser de 0,54, demonstrando que seria improvável que a concordância pelos juízes tivesse se dado ao acaso.

Na ***terceira etapa*** , as palavras consideradas familiares, ou seja, as essenciais, conforme o cálculo da RVC, formaram um banco de palavras que foram utilizadas na elaboração da versão preliminar das novas listas de monossílabos, constituída de cinco listas com 25 monossílabos em cada lista, denominadas listas L1, L2, L3, L4 e L5, que foram gravadas digitalmente, conforme a norma ISO 8253-3:2012.

Na ***quarta etapa*** , foram selecionados por conveniência, 40 indivíduos com idades entre 18 e 44 anos, destros, de diferentes escolaridades, normo-ouvintes (limiares auditivos ≤ 25 dB NA, nas frequências de 250 Hz a 8.000 Hz, conforme audiometria tonal liminar), sem alterações no meato acústico externo, e de orelha média, a partir da inspeção visual e curva timpanométrica, respectivamente, ou ainda sem outros comprometimentos, como alteração de compreensão e/ou emissão oral. Aqueles sujeitos que preencheram os critérios de seleção e que assinaram o TCLE, participaram da pesquisa.

Tais indivíduos foram considerados juízes ouvintes, para os quais foram apresentadas as listas de palavras, tendo sido solicitado, como resposta, que repetissem as palavras da forma que entendessem. O nível de apresentação foi de 40 dB NS.

Na ***quinta* e última etapa**, foi realizado o julgamento auditivo das palavras, por meio de análise descritiva e qualitativa dos erros produzidos pelos indivíduos. Ainda, foram comparados os erros apresentados por orelha, utilizando o teste U de Mann-Whitney (duas amostras independentes, teste não paramétrico), e também considerando o nível de escolaridade, utilizando o teste Kruskal-Wallis (quatro amostras independentes, teste não paramétrico). Foi adotado um intervalo de confiança de 95%.

Após esta etapa, houve uma reorganização das palavras nas listas. Nesta reorganização, foram excluídos os vocábulos emitidos de forma incorreta por mais de um sujeito, sendo estes considerados erros não aleatórios, e também, foi excluída a quinta lista devido à redução no número de vocábulos disponíveis e por apresentar a composição mais heterogênea. Assim, restaram quatro listas, com 25 monossílabos validados quanto ao conteúdo, prontas para serem avaliadas quanto à equivalência entre si.

### Pesquisa de equivalência das listas

Após a validação de conteúdo das listas de monossílabos, estas foram submetidas à pesquisa de equivalência.

Nesta etapa, foram selecionados por conveniência 60 indivíduos, com idades entre 18 e 24 anos, considerando os demais critérios de inclusão citados na quarta etapa.

Neste trabalho, optou-se por utilizar um ruído fixo, do tipo *speech noise*, na intensidade de 30 dB NA, a fim de nivelar a audição de todos os participantes, e também neutralizar qualquer possível interferência de ruído corporal ou ambiental que pudesse interferir no desempenho dos indivíduos avaliados, considerando também um nível de apresentação que não provocasse desconforto e/ou cansaço. Esta estratégia de utilização de um ruído fixo, para verificação de equivalência de listas de monossílabos, já havia sido aplicada por outros pesquisadores^(14)^.

A partir desse momento, foi realizado um estudo piloto, procurando determinar a relação sinal/ruído (S/R) que permitiria obter escores de reconhecimento que variassem em torno de 40 e 60%. Esta estratégia foi utilizada com a finalidade de diminuir a variabilidade do desempenho dos indivíduos avaliados, além de evitar o “efeito floor” ou o “efeito celling”^(15)^, que ocorre quando o desempenho é de 0% ou 100%.

Dessa forma, considerando as questões referidas acima, para o estudo piloto, as listas de palavras foram apresentadas na presença de um ruído fixo, apresentado ipsilateralmente (fala e ruído na mesma orelha) em diferentes relações S/R, e a relação de -1 dB NA foi a condição na qual os escores mais se aproximaram da faixa esperada.

A partir do estudo piloto, a pesquisa de equivalência das listas foi realizada com base no desempenho dos demais indivíduos, apresentando as palavras no nível de 29 dB NA e ruído a 30 dB NA (relação S/R de -1 dB). A ordem de apresentação das listas foi randomizada, sendo que 30 deles ouviram os vocábulos na orelha direita e os outros 30, na orelha esquerda.

As respostas dos sujeitos para cada palavra foram consideradas: incorretas, atribuindo-se o valor de “0” ou corretas, o valor de “1”. A partir desses valores, obteve-se o percentual de acertos das palavras por lista, indicando a variabilidade dentro de cada lista. Também foi considerado o escore total do sujeito por lista, multiplicando-se por quatro, o número de acertos de cada sujeito, para se obter o índice percentual do reconhecimento das palavras, ou seja, o desempenho do sujeito por lista.

Para analisar o desempenho apresentado pelos sujeitos segundo o lado da orelha, foi utilizado o teste de Kruskal-Wallis (variáveis com distribuição não normal, teste não paramétrico).

A seguir, foram analisados os percentuais de acertos das palavras por lista elaborada, para a obtenção do percentual de inteligibilidade de cada lista, e então, realizada a análise da variância das palavras por lista, aplicando-se o teste ANOVA (variáveis com distribuição normal, teste paramétrico).

Por fim, foram analisados os escores dos sujeitos por lista, e por serem variáveis com distribuição não normal, foram usados testes não paramétricos: teste de Friedman e a análise da variância entre as listas, e para identificar as diferenças entre as listas, foi utilizado o teste de Wilcoxon. Também foi realizada análise estatística descritiva de algumas variáveis, para complementar a análise dos dados.

Adotou-se um nível de significância de 5% (p-valor ≤ 0,05). Os resultados estatisticamente significantes foram assinalados com um (*). As análises estatísticas foram realizadas pelo *software Statistica 9.1.*

## RESULTADOS

Quanto à validação de conteúdo, a análise dos julgamentos da familiaridade dos 125 vocábulos selecionados, com base no cálculo da Razão de Validade de Conteúdo (RVC), revelou que todas as palavras apresentaram RVC ≥ 0,529, sendo julgadas essenciais (extremamente familiar, muito familiar e familiar), não havendo necessidade de exclusão de nenhum vocábulo nesta etapa (Tabela 1).

**Tabela 1:** Distribuição dos monossílabos essenciais (n=125) nas listas elaboradas com base no Cálculo da Razão de Validade de Conteúdo

**N Palavras (%)**	**N avaliadores** **(N, %)**	**RVC**
**59 (47,2%)**	17 (100%)	1
**25 (20%)**	16 (94,11%)	0,882
**12 (9,6%)**	15 (88,23%)	0,764
**04 (3,2%)**	14 (82,35%)	0,647
**25 (20%)**	13 (76,47%)	0,529

Legenda: N = número; RVC = Razão de Validade de Conteúdo

Em relação aos erros produzidos pelos sujeitos ao realizarem o reconhecimento auditivo dos vocábulos, observa-se que não houve diferença estatisticamente significante, ao comparar os erros apresentados pelos sujeitos que ouviram as palavras na orelha direita, com os que ouviram as palavras na orelha esquerda, o que permitiu que os dados fossem agrupados para análise apenas dos erros produzidos pelos sujeitos (Quadro 1).

**Quadro 1:** Reconhecimento auditivo das listas de monossílabos por sujeitos de diferentes escolaridades

Escolaridade	N	Palavras produzidas com erro					N de erros por orelha		p-valor	N de palavras com erro por escolaridade	p-valor	N de sujeitos com erro no reconhecimento
		L1	L2	L3	L4	L5	OD	OE				
Ensino Superior	10	0	bem	0	0	0	0	1	0,735	1	0,113	1
Ensino Médio	10	giz	tom	deu	0	ai	2	2		4		4
Fundamental Completo	10	for	viu	0	0	0	2	0		2		2
Fundamental Incompleto	10	giz	sei	0	pai	ai zaz	3	2		5		4
Total	40	3	4	1	1	3	7	5		12		11

Análise dos erros por orelha: Teste U de Mann-Whitney (p-valor = 0,735)

Análise dos erros por escolaridade: Teste Kruskal-Wallis (p-valor = 0,113)

Valor estatisticamente significante (p-valor ≤0,05)

Legenda: L1 = Lista 1; L2 = Lista 2; L3 = Lista 3; L4 = Lista 4; L5 = Lista 5; OD = orelha direita; OE = orelha esquerda; N = número

A validação de conteúdo resultou em quatro listas de monossílabos (Figura 1). Observa-se que a maior parte dos fonemas que compõem as listas está representada pelas vogais e glides e também pelo fonema fricativo /s/ que esteve presente em várias posições nas sílabas. Não há, em nenhuma das listas, monossílabos com os fonemas /ɲ/, /ʎ/ e /z/, no entanto, as classes desses fonemas estão representadas pelos fonemas /m/ e /n/, /l/ e /s/, respectivamente.

**Figura 1:** Apresentação da versão final das listas de monossílabos elaboradas e validadas quanto ao conteúdo - distribuição dos fonemas por lista e relação dos moldes silábicos

Legenda: L1 = Lista 1; L2 = Lista 2; L3 = Lista 3; L4 = Lista 4

No que se refere à pesquisa de equivalência das listas, foi possível observar por meio do teste de Kruskall-Wallis, que os valores de p (p = 1,000 para todas as análises realizadas) obtidos pela comparação dos desempenhos apresentados pelos sujeitos, nas diferentes listas, por orelha, não foram estatisticamente significantes, o que mostrou não haver diferença de desempenho segundo o lado da orelha, e assim as seguintes análises das respostas dos sujeitos foram realizadas conjuntamente.

Sobre as medidas descritivas referentes aos percentuais de acerto (Tabela 2), de um lado, estão representados os percentuais de inteligibilidade das palavras por lista, analisando a variabilidade intralista, do outro, estão representados o desempenho dos sujeitos no reconhecimento das palavras por lista de monossílabos, analisando a variabilidade interlistas.

**Tabela 2:** Análise descritiva dos dados, expressa em percentual de acertos, apresentando os resultados de acordo com a variabilidade intralista e interlistas

**Análise das palavras por Lista**				**Lista**	**Desempenho dos sujeitos por Lista**			
L1	L2	L3	L4		L1	L2	L3	L4
25	25	25	25	**N**	60	60	60	60
48,33	48,00	55,73	51,93	**Média (%)**	48,33	48,00	55,73	51,93
45	50	60	50	**Mediana (%)**	48	48	56	52
1,66	8,33	10	8,33	**Mínimo (%)**	36	32	44	40
98,33	91,66	96,66	95,00	**Máximo (%)**	64	68	72	72
28,33	25,00	36,66	31,66	**Quartil Inferior (%)**	44	44	52	44
70,00	61,66	75,00	75,00	**Quartil Superior (%)**	52	52	60	56
29,545	26,369	25,088	27,565	**Desvio-padrão (%)**	7,036	6,628	6,744	7,741

Desta forma, considerando-se o percentual de acertos das palavras nas listas, percebe-se que a lista L3 apresentou a maior média de acertos, parecendo ser uma lista mais fácil. Ainda, observa-se que a média do percentual de acertos das palavras por lista é muito semelhante nas listas L1 e L2, indicando que estas seriam as listas mais homogêneas. Já as listas L3 e L4 apresentam, uma média diferente das listas L1 e L2. No entanto, não foi apontada nenhuma diferença estatisticamente significante nesse sentido resultante da realização do teste ANOVA (p-valor = 0,722).

Por sua vez, ao analisar os resultados da comparação dos escores dos sujeitos entre as listas de monossílabos, os quais mostram a mediana do percentual de acertos dos sujeitos por lista, é possível identificar visualmente a semelhança existente entre as listas L1 e L2, ao mesmo tempo em que se observa uma pequena semelhança entre L1 e L2 com L4, muita diferença entre L1 e L2 em relação à L3 e também uma pequena semelhança entre L3 e L4 (Figura 2). O teste de Friedman apontou diferença estatística entre as listas, com base no desempenho dos sujeitos.

**Figura 2:** Representação da comparação entre o desempenho dos sujeitos no reconhecimento dos monossílabos das listas L1, L2, L3 e L4. Representação da mediana e dos valores mínimo e máximo em relação ao percentual de acerto dos sujeitos por lista de monossílabos - Variabilidade interlistas Teste de Friedman – múltiplas amostras dependentes, pareado por sujeito; p-valor = 0,00000* *Valor estatisticamente significante (p-valor ≤ 0,05) Legenda: Median = Mediana; Min = Mínimo; Max = Máximo

Ainda, o resultado da análise estatística com a aplicação do teste de Wilcoxon evidencia que a lista L1 não difere da L2, e que houve diferença estatisticamente significante ao comparar estas com as listas L3 e L4 e, as listas L3 e L4 entre si (Tabela 3).

**Tabela 3:** Comparação entre as listas de monossílabos com base no desempenho dos sujeitos no reconhecimento das palavras por lista

**Listas**	**p-valor**
L1 = L2	0,776
L1 ≠ L3; L4	≤ 0,01*
L2 ≠ L3; L4	≤ 0,001*
L3 ≠ L4	≤ 0,003*

Teste de Wilcoxon – amostras dependentes

* Valor estatisticamente significante (p-valor ≤ 0,01)

## DISCUSSÃO

A validação de conteúdo é uma etapa essencial no processo de construção e adaptação de instrumentos de medidas^(13)^. Está relacionada ao planejamento do teste, momento no qual se organiza um conjunto de itens relevantes e representativos do conteúdo que se quer avaliar. Assim, em relação aos testes de fala, é necessário considerar, inicialmente, a seleção das palavras, pois estas apresentam particularidades próprias de cada língua, devendo ser padronizadas e testadas nos sujeitos^(2)^.

As estruturas silábicas das 125 palavras selecionadas para o instrumento proposto neste estudo, descritas na metodologia, estão de acordo com as características da Língua Portuguesa, mantendo as estruturas silábicas próprias da língua. Um levantamento da frequência de ocorrência dos padrões silábicos do Português Brasileiro, independente do número de sílabas das palavras, obtido com base em palavras escritas de um dicionário, aponta que a estrutura CV é a mais frequente na língua, seguida pelas sílabas de tipo CVG ou CVC, V, VC e CCV, sendo as sílabas CCVC e CCVCC, menos frequentes^(16,17)^. Também deve-se considerar que na Língua Portuguesa, há um número restrito de monossílabos^(17)^, sendo, portanto, poucas combinações de consoante e vogal que produzem palavras com significado.

Neste estudo, a inclusão das palavras nas listas elaboradas priorizou a familiaridade das mesmas, em detrimento do balanceamento fonético (Tabela 1), pois segundo a literatura^(18)^, a familiaridade de um vocábulo é dependente da frequência de uso na língua e também está relacionada com a melhora na inteligibilidade das palavras, além do que, a escolha de palavras familiares minimizaria o efeito das diferenças educacionais entre os sujeitos testados, justificando e reforçando assim o critério escolhido para a seleção das palavras.

Ainda, ao escolher as palavras para formar as listas para este estudo, manteve-se uma amostra representativa dos sons da língua, com uma distribuição dos fonemas entre as listas, o mais uniforme possível. Esta prática foi corroborada na literatura consultada, evidenciando-se que o balanceamento fonético é de importância secundária, pois os vocábulos mais usados e significantes são aqueles que permitem melhor reconhecimento de fala^(7,19)^, reforçando mais uma vez a necessidade de inclusão de palavras familiares.

Dos testes de fala elaborados no Brasil, com palavras monossilábicas, Sá^(8)^ elaborou listas balanceadas foneticamente. Lacerda^(10)^ escolheu palavras cujo material fonético fosse representativo da língua, sem, no entanto, realizar um rigoroso balanceamento fonético. Ainda, cuidou para que as palavras fossem familiares, que fizessem parte do vocabulário usual, que representassem substantivos concretos, que fossem acessíveis a pessoas de diferentes níveis culturais e que as listas tivessem o mesmo grau de dificuldade entre si.

Pen e Mangabeira-Albernaz^(9)^ e Russo e Santos^(5)^ usaram palavras familiares, mas não esclareceram como selecionaram as palavras. Chaves et al.^(11)^ e Roll et al.^(4)^ se basearam nos critérios propostos por Lacerda^(10)^ para escolha das palavras. Chaves et al.^(11)^ elaboraram listas de 25 palavras e 25 pseudopalavras (monossilábicas e dissilábicas) e Roll et al.^(4)^ elaboraram duas listas de monossílabos, sendo uma lista com 25 e outra com 50 itens.

Ao analisar a influência da escolaridade nos erros produzidos pelos sujeitos avaliados (Quadro 1), não foi comprovada diferença estatisticamente significante, porém foi constatado que 11 sujeitos apresentaram erros no reconhecimento auditivo de 12 palavras.

A maioria dos erros apresentados pelos sujeitos foi considerado como sendo ao acaso, pois foram erros isolados. No entanto, alguns podem ser explicados pelas características acústico-articulatórias dos fonemas e também pela familiaridade, pelo significado que a palavra assume para o ouvinte, conforme referem alguns autores^(4,20)^.

Também, observa-se que as trocas foram mais frequentes nos sons consonantais do que com as vogais, o que se assemelha aos resultados obtidos em outro estudo^(21)^, justificando que a inteligibilidade de fala é dependente dos sons consonantais, que representam uma contribuição de 60%, enquanto as vogais, contribuem apenas 40%.

As pesquisas envolvendo a equivalência de listas de monossílabos apresentam estratégias muito variadas. Sendo assim, na análise da equivalência das listas de monossílabos do Mandarin^(22)^, por exemplo, os autores fixaram o nível do sinal da fala em 10 dB NS a partir da média tritonal das frequências de 500, 1000 e 2000 Hz, para obter escores de reconhecimento entre 40 e 60%. Também, em outra pesquisa com listas de monossílabos do Mandarin^(14)^, cada sujeito foi testado com todas as listas sendo aplicadas em sequências diferentes e com diferentes níveis de apresentação (-5, 0, 5, 10 e 15 dB NA). Outro estudo, que investigou as funções psicométricas na presença de ruído^(23)^, utilizou quatro relações S/R (-7, -2, 3 e 8 dB), mantendo o ruído fixo a 72 dB SPL, buscando o ponto de 50%. Estes pesquisadores, cada um com suas estratégias, conseguiram avaliar a equivalência das suas listas, e confirmar que o desempenho dos sujeitos sofria importante influência da relação S/R escolhida.

Dessa forma, por não termos encontrado na literatura consultada nenhuma estratégia que fosse consenso, e por se tratar de um novo material, foi utilizada para a verificação da equivalência entre as listas desta pesquisa, a apresentação das listas de palavras na presença de diferentes níveis de ruído, em um estudo piloto, o que permitiu determinar a relação S/R adequada para pesquisar o índice de reconhecimento de fala evitando a ocorrência do efeito “*floor”* ou o efeito “*ceiling*”^(15)^. Estes efeitos devem ser evitados, pois provocam a perda de informações nos dados observados, em função da possibilidade das pontuações verdadeiras estarem acima dos limites mínimos ou máximos observadas no limiar, onde não é mais possível mensurar a variável, sendo estes considerados dados censurados, os quais são parcialmente conhecidos^(24)^.

Acredita-se que a variedade de estratégias utilizadas pelos diferentes pesquisadores, não seja um problema, pois sabe-se que muitos fatores estão envolvidos neste tipo de análise, tais como: equipamento usado, calibração, forma de aplicação, composição e familiaridade dos vocábulos, voz do locutor, tipo de ruído a ser utilizado, características dos indivíduos avaliados, entre outros. Salienta-se que nesta fase da pesquisa, o que se buscou foi estabelecer uma medida relativa, que consistiu na comparação das diferentes listas entre si, mantendo as condições de testes invariáveis e características individuais muito semelhantes dos sujeitos avaliados, com critérios de inclusão e exclusão rígidos, tendo como variável apenas os sujeitos, evitando ao máximo comprometer os resultados obtidos.

A variabilidade intralista foi expressa neste estudo por meio do percentual de inteligibilidade de cada palavra por lista (Tabela 2), fornecendo, assim, informações sobre a variação da inteligibilidade dos itens que compuseram as listas.

Observa-se que nas quatro listas, nenhuma das palavras apresentou 0% ou 100% de acertos, havendo, estatisticamente, um equilíbrio entre a inteligibilidade das palavras de cada lista. Este resultado está de acordo com os pressupostos acerca dos critérios a serem observados na elaboração dos materiais de fala^(25)^.

Neste estudo, percebe-se que as listas L1 e L2 são muito semelhantes (Tabela 2 e Figura 2), confirmado pelos valores de média e mediana, que representam uma medida de tendência central para os escores de reconhecimento de fala apresentados pelos sujeitos. Os valores encontrados nos quartis também sugerem que as listas L1 e L2 são equivalentes. A lista L4 foi a que apresentou o terceiro valor para média e mediana, sendo semelhante às listas L1 e L2, mas não equivalente a elas. A lista L3, foi considerada a lista mais fácil dentre as quatro listas propostas.

É importante salientar que a obtenção da equivalência de diferentes listas de palavras é uma tarefa complexa, e difícil de ser obtida mesmo com todos os critérios embasados na literatura para a seleção das palavras, elaboração das listas e gravação das mesmas^(2,19,25)^, que pode ser atribuído a uma série de fatores, que estão relacionados à inteligibilidade dos vocábulos das listas que englobam desde a construção fonética, a familiaridade da palavra, o ambiente fonético da palavra e, principalmente, as características de gravação dos testes de fala, incluindo as técnicas de gravação e as características do locutor^(25,26)^.

Considerando os resultados obtidos (Figura 2 e Tabela 3), foi observado que a lista L4, apesar de não apresentar equivalência estatística com as Listas L1 e L2, possibilitou escores semelhantes e apresentou homogeneidade em relação à variação da inteligibilidade dos itens. Assim, sugere-se que a mesma seja utilizada como uma lista-treino, o que é extremamente útil a fim de familiarizar o indivíduo com o teste, quando for necessário.

A lista L3 será excluída, apesar de ter sido considerada homogênea em relação à variação na inteligibilidade dos itens, pois apresentou escores muito diferentes em relação às demais listas.

Após a validação de conteúdo e pesquisa de equivalência, as listas oriundas deste trabalho foram renomeadas em ordem alfabética e, assim, denominadas Lista Treino, L1 e L2 (Apêndice 1).

Salienta-se que as medidas psicométricas obtidas neste estudo são válidas para as listas gravadas como elas estão. Se forem regravadas por outra pessoa ou até mesmo, pela mesma pessoa, ou se forem apresentadas à viva voz, ou ainda, se houver uma nova organização das palavras originando outras listas, novas medidas psicométricas precisarão ser obtidas, sendo esta premissa corroborada na literatura consultada^(23,27,28)^.

Acredita-se que a elaboração e validação do material proposto, em formato digital, conforme recomendações da literatura, permitirá a realização do IPRF de maneira mais precisa, diminuindo a variabilidade e aumentando a reprodutibilidade dos resultados obtidos na avaliação à viva voz.

## CONCLUSÃO

Duas listas de monossílabos foram validadas quanto ao conteúdo e consideradas equivalentes e uma lista foi considerada semelhante, podendo ser utilizada como treino, formando um conjunto de três listas desenvolvidas na Língua Portuguesa Brasileira, gravadas digitalmente, que poderão fazer parte da bateria logoaudiométrica para serem aplicadas na rotina clínica e em pesquisas, a partir da sua validação e publicação. As listas validadas não sofreram influência segundo o lado da orelha e escolaridade.

## Footnotes

1 Nesse molde silábico foram considerados como glide as consoantes nasal e líquida lateral na posição de coda final e a segunda vogal dos ditongos orais e nasais, devido às suas características fonéticas.

2 As consoantes do final da sílaba, na posição de coda final, estão representadas pelas consoantes /r/ e /s/.

## Apêndice 1 Listas de monossílabos para testes logoaudiométricos

**Table:**

	LISTA TREINO	L1	L2
1	não	bar	dez
2	só	dó	fim
3	véu	eu	pá
4	paz	for	ver
5	lei	gás	mas
6	dom	mil	dói
7	fã	céu	seu
8	eu	vão	lã
9	bis	ter	gel
10	rio	jaz	rês
11	mar	nem	chão
12	pé	pós	nó
13	gol	dei	sei
14	foz	tão	por
15	cão	som	mau
16	ser	rói	fiz
17	já	leu	tom
18	meu	voz	viu
19	xis	cai	eu
20	tem	mim	cós
21	dor	pau	mão
22	sal	sã	réu
23	rim	ré	lar
24	pai	lá	sai
25	vez	fez	bem
